# Development of Poly (Lactide Acid) Foams with Thermally Expandable Microspheres

**DOI:** 10.3390/polym12020463

**Published:** 2020-02-17

**Authors:** Ákos Kmetty, Katalin Litauszki

**Affiliations:** 1 Department of Polymer Engineering, Faculty of Mechanical Engineering, Budapest University of Technology and Economics, Műegyetem rkp. 3., H-1111 Budapest, Hungary; litauszkik@pt.bme.hu; 2 MTA–BME Research Group for Composite Science and Technology, Műegyetem rkp. 3., H-1111 Budapest, Hungary

**Keywords:** thermally expandable microspheres, physical blowing agent, poly (lactic acid) foam, d-lactide, foam extrusion, syntactic foam structure

## Abstract

This study presents the investigation of different content of thermally expandable microsphere (EMS) type of a physical blowing agent added to polylactic acid (PLA). The effects of the different doses of EMS, processing temperatures, and d-lactide content of the polylactic acid were analyzed for foam properties and structures. We characterized the different PLAs and the physical blowing agent with different testing methods (gel permeation chromatography, rotational rheometry, isothermal thermogravimetric analysis, and thermomechanical analysis). The amounts of the foaming agent were 0.5, 1, 2, 4, 8 wt%, and processing temperatures were 190 °C, 210 °C, and 230 °C. The foam structures were produced by twin-screw extrusion. We used scanning electron microscopy to examine the cell structure of the foams produced, and carried out morphological and mechanical tests as well. The result of extrusion foaming of PLA using different amounts of EMS shows that an exponentially decreasing tendency of density reduction can be achieved, described by the following equation, *ρ*(*x*$)=1.062\cdot e^{-\frac{x}{7.038}}+0.03$ (R^2^ = 0.947) at 190 °C. With increasing processing temperature, density decreases at a lower rate, due to the effect that the microspheres are unable to hold the pentane gas within the polymer shell structure. The d-lactide content of the PLAs does not have a significant effect on the density of the produced foam structures.

## 1. Introduction

The foaming of polymers is possible mechanically (air dispersion), physically (gas injection, bead foaming, expandable microspheres), and chemically (foaming agents that generate effective gases through thermal decomposition, e.g., in the case of during polyurethane foaming) [1,2,3]. Nowadays, the polymer foam market is growing at an annual rate of about 3–6% [4], mainly because polymer foams have low weight and excellent thermal, acoustic, moisture insulating, and damping properties [5,6,7,8,9,10,11]. Due to the increasingly stringent environmental directives and the reduction/prohibition of single-use products, polylactide acid (PLA) is one of the most important biopolymer candidates to enhance sustainability. Due to PLA’s low melt elasticity [12], melt strength [13], and slow crystallization kinetics [14], PLA extrusion foaming is challenging and has its limitations. The foam density achieved with the use of chemical foaming agents (CBA) during extrusion is higher than 0.5 g/cm^3^. For extrusion foaming of PLA with a physical blowing agent, density reduction can be made most successfully with CO_2_ [15]. The resulting density range is lower than 0.1 g/cm^3^, although for this, both the PLA raw material (e.g., with a chain-extender [16], a nucleating agent [17], or by post-production heat-treatment [18]) and the processing equipment need to be modified [19].

One way of producing high and medium-density (>0.1 g/cm^3^) polymeric foam compositions is to use a special physical foaming agent and syntactic foam forming, extrusion, injection molding, or even rotational molding. This foaming agent expands during processing when added to a given polymeric matrix material (either thermoplastic or thermoset). Initially, beads with an average diameter of ~10 µm can expand up to 10 times their diameter. The microspheres have a thermoplastic shell layer, within which a low boiling point hydrocarbon (e.g., pentane) provides the expansion over a given temperature range. They can be used to create spherical cavities (closed cells) in a given polymer matrix material under controlled conditions in a more reproducible manner than chemical foaming agents. So far, microspheres have been used mainly for the foaming of polypropylene [20], thermoplastic polyurethane [21], and polyvinyl chloride [22], polyester [23], and epoxy resin [24]. They are barely used in renewable resource-based and/or biodegradable polymer-based polymers [25]. Our goal is to produce PLA-based foam structures via extrusion exceeding the already available density reduction of chemically foamed PLA foams, and to analyze morphologically, mechanically, and microscopically renewable resource-based and biodegradable-based (e.g., composting) polylactic acid foam structures foamed with expandable microspheres, as these can be alternative environmental friendly foam structures.

## 2. Materials and Methods

We used different polylactic acid grades from NatureWorks© LLC, Minnetonka, MN, USA Ingeo 4032D, 2003D and 4060D (1.4, 4.3 and 12.0 mol% d-lactide content [26]), with a density of 1.24 g/cm^3^ [27]. The PLAs have a melting temperature (T_m_) of 169 °C, 151 °C, and in the case of amorphous PLA with 12.0% d-lactide content, there was no detectable T_m_. We performed a DSC test, and from the first melting curve, (5 °C/min), we obtained a Melt Flow Index of 2.5, 2.0, and 3.2 g/10 min (CEAST 7027.000, 2.16 kg, at 190, 210, and 230 °C). The degree of crystallinity was 40.6% (4032D), 28.4% (2003D), and 0% (4060D), as measured by the authors [28].

We used thermally expandable microsphere (EMS) physical blowing agent (PBA) with different amounts (0.5, 1, 2, 4, 8 wt%) for the extrusion foaming of PLA. The type of the EMS is Tracel G 6800 MS (Tramaco GmbH, Tornesch, Germany). The carrier polymer of the foaming agent is ethylene-vinyl acetate copolymer (EVA). The shell structure of the expandable microspheres contains methyl methacrylate (MA), acrylonitrile (AN), and or methacrylonitrile (MAN), as well as silicon dioxide (SiO_2_). The effective gas is isopentane. Thermogravimetric analysis (TGA) was performed on the EMS foaming agent to determine the component ratios, i.e., the amount of effective gas, the ratio of the polymeric carrier and the expandable microspheres. The effective gas (isopentane) was 18.6%, the shell structure of EMS (acrylonitrile/methyl methacrylate) was 16%, the carrier material (EVA) was 36.8%, and the silica stabilizer with a weight ratio of 25%, previously measured by the authors [28]. The amount of yielded gas was calculated based on the weight loss percentage of the foaming agent, determined by TGA. The calculated value was 24 mL/g.

### 2.1. Gel Permeation Chromatography of PLA

To measure the PLA samples number-average molecular weight ($\bar{M_{n}}$) and weight average molecular weight ($\bar{M_{w}}$), we used gel permeation chromatography (Agilent PL-GPC 50 System, Santa Clara, CA, USA). The samples were dissolved in chloroform and filtered through a PTFE filter. Four Agilent PL-Gel columns at 30 °C (3 x PL-Gel Mixed C (5 μm) and 1 x PL-Gel Mixed E (3 μm) columns) were used in series, with amylene-stabilized HPLC grade chloroform as the eluent (flow rate of 1 mL/(1/min)), on a Waters Alliance system equipped with an Alliance 2695 Separation Module. Polymer number-average molecular weight ($\bar{M_{n}}$) and polydispersity index ($\bar{M_{w}}$/$\bar{M_{n}}$; PDI) were calibrated against low dispersity polystyrene standards with a 3rd order polynomial fit, linear across molar mass ranges.

### 2.2. Rotational Rheometry of PLA

PLA matrix materials were characterized with an AR2000 type of rotational rheometer (TA Instruments, New Castle, DE, USA). The tested specimens (Ø25 x 1 mm dimensions) were produced by hot pressing at 190 °C with 100 bar. The viscosity curves were represented as a function of strain rate. We used the following parameters: Test temperatures were 190 °C, 210 °C, and 230 °C, test frequencies ranged from 1–100 Hz, deformation rate was 10%, and the type of plate was parallel aluminum plates with 25 mm diameters. To convert oscillatory data to steady shear data, we used the Cox-Merz rule [29].

### 2.3. Thermogravimetric Analysis Performed in Isothermal Conditions of EMS

Thermogravimetric analysis (TGA) was performed with a TA Instruments (New Castle, DE, USA) Q500. The test temperatures were 190 °C, 210 °C, and 230 °C, the heating rate was 100 °C/min, the mass of the samples was around 1 mg. The amount of yielded gas was calculated based on the weight loss percentage of the foaming agent, determined by TGA. The tests were performed in protective nitrogen gas (40 mL/min) and industrial air atmosphere (60 mL/min). These conditions are closer to those in an extruder.

### 2.4. Thermomechanical Analysis of EMS

Thermomechanical analysis (TMA) was performed with a TA Instruments Q400 (New Castle, DE, USA) to characterize the expansion of EMS. The measured temperature range was 25–250 °C, the heating rate was 20 °C/min and nitrogen gas was applied as a measuring atmosphere (gas flow: 50 mL/min). As a preload force, 0.06 N was set.

### 2.5. Differential Scanning Calorimetry of Biopolymer Foams

Differential scanning calorimetry (New Castle, DE, DSC) tests of biopolymer foams were carried out with a TA Instruments (New Castle, DE, USA) Q2000. The temperature range was 0–200 °C, the heating rate was 5 °C/min, the mass of the samples was between 3 mg and 6 mg, and the tests were performed in a nitrogen atmosphere (50 mL/min). The degree of crystallinity ($\chi_{c}$) was calculated according to Equation (1), where $\Delta H_{m}$ is the melting enthalpy. The degree of crystallinity ($\chi_{cf}$) created via foam processing was calculated according to Equation (2), where $\Delta H_{m}$ is the melting and $\Delta H_{cc}$ is the cold crystallization enthalpy. PLA_100%_ is the theoretical melting enthalpy of 100% crystalline PLA, which is 93 J/g [30].

(1) $$\chi_{c}=\frac{H_{m}}{{\mathrm{PLA}}_{100\%}}\cdot 100\;\left[\%\right],$$

(2) $$\chi_{cf}=\frac{H_{m}-\left|H_{\mathrm{cc}}\right|}{{\mathrm{PLA}}_{100\%}}\cdot 100\;\left[\%\right],$$

### 2.6. Scanning Electron Microscopy

The cell structures were analyzed by scanning electron microscopy (SEM) with a JEOL (Tokyo, Japan) JSM 6380LA machine with an accelerating voltage of 10 kV. The cryogenic fracture surface of the samples was pre-coated with a gold-palladium alloy with JEOL 1200 (Tokyo, Japan) and fixed with a conductive double-sided carbon adhesive tape.

### 2.7. Foam Characterization

The density of the foam structures and solid materials was calculated with Equation (3). The weight of the samples was measured with an Ohaus Explorer (Nänikon, Switzerland) analytical balance. Measurement accuracy was 0.0001 g. The medium was distilled water.

(3) $$\rho =\frac{m_{sa}}{m_{sa}-m_{sl}}\cdot \rho_{dw},$$

where *ρ* (g/cm^3^) is the density of the measured specimens; *m_sa_* (g) is the mass derived from the weight of the specimen in air; *m_sl_* [g] is the mass derived from the weight of the specimen measured in the measuring fluid, *ρ_dw_* [g/cm^3^] is the density of the measuring medium. The void fraction was calculated according to Equation (4) [31].

(4) $$V_{f}=1-\frac{\rho_{\mathrm{foam}}}{\rho_{\mathrm{polymer}}},$$

where *V_f_* [–] is the void fraction, $\rho_{foam}$ [g/cm^3^] is the density of the foamed polymer, and $\rho_{\mathrm{polymer}}$ [g/cm^3^] is the density of the non-foamed polymer. Cell population density was calculated based on the SEM images, according to Equation (5), where *n* is the number of cells counted in the recorded image, *A* [cm^2^] is the cross-section area of the sample, *M* [–] is the magnification factor, and *V_f_* [–] is the void fraction [31].

(5) $$N_{c}={\left(\frac{{n*M}^{2}}{A}\right)}^{\frac{3}{2}}\cdot \;\frac{1}{1-V_{f}},$$

The theoretical value of density was calculated with Equation (6), where *kg components* is the mass of the material used in the formula, and *kg components* divided by the *avg density of the components* represents the volume of the material [32]. The amount of yielded gas was calculated based on the weight loss percentage of the foaming agent, determined by TGA.

(6) $$\mathrm{Theoretical}\;\mathrm{density}=\frac{\left(\mathrm{kg}\;\mathrm{components}\right)\;}{\frac{\;\left(kg\;components\right)}{avg\;density\;of\;components)}+volume\;of\;gas}.$$

### 2.8. Compression Strength

Foam compression strength tests were performed with a Zwick Z005 (Ulm, Germany) testing machine. The type of the load cell was Mess and Regeltechnik KAP-TC (Dresden, Germany) with measuring range 0–5000 N, preload 1 N. The test speed was 2 mm/min. Specific foam compressive strength was calculated per Equation (7).

(7) $$\sigma =\frac{\frac{F_{10\%}}{A_{\mathrm{foam}}}}{\rho },$$

where *specific compressive strength* [MPa/(g/cm^3^)] is the compression strength at 10% deformation, $F_{10\%}$ [N] is the force at 10% deformation, *A_foam_* [mm^2^] is the cross-sectional area of the foam specimen and *ρ* [g/cm^3^] is the density of the foam sample.

### 2.9. Dynamic Mechanical Analysis

Dynamic mechanical analysis (DMA) was performed on a TA Instruments Q800 (New Castle, DE, USA) instrument. Before the test, the linear viscoelastic behavior of the sample was checked. The dimensions of the specimens were 10 mm × 60 mm. Dual cantilever clamping was used with a 35 mm width support. After 5 min of holding time, the test was carried out in a temperature range of 0 to 150 °C, with a heating rate of 2 °C/min. The amplitude of the periodic bending stress was 20 μm and its frequency was 1 Hz.

### 2.10. Extrusion Foaming of PLA (Rod-Shaped Specimen)

Foaming was carried out with a Teach-line ZK25T (Collin GmbH, Ebersberg, Maitenbeth, Germany) counter-rotating twin-screw extruder (screw diameter: 25 mm, L/D = 24). Three processing temperature profiles were used. Table 1 shows the temperature profiles of the five zones starting from the hopper (Z1). The screw speed was 10 rpm. Rod type of extrusion die was used with a circular cross-section (d = 3 mm). The EMS foaming agent was added to the PLA granules before extrusion by dry mixing. Before manufacturing, the crystalline PLA granules were dried at 80 °C for 6 h, and in the case of amorphous PLA, at 45 °C for 8 h in a WGL-45B type drying oven.

### 2.11. Extrusion Foaming of PLA (Sheet Specimen)

Based on the results of manufacturing rod-shaped foamed products, we conducted an adaptability study for the production of sheet-shaped products of Ingeo 4060D PLA. A flat sheet production line was used consisting of an extruder (Labtech 25-30C (Labtech Engineering Co., Ltd., Samutprakarn, Thailand)) and a temperature-controlled and polished roller attachment unit with pneumatic drawing (Labtech LRC300 (Labtech Engineering Co., Ltd., Samutprakarn, Thailand)). The temperature profile used in sheet extrusion was the same as the first profile in Table 1 (155/160/175/190/190 °C), and the temperature of the adapter and die was 190 °C. The adjustable gap of the coat-hanger die was 4 mm long and 300 mm wide. The screw speed was 80 rpm to allow the polymer to build up suitable processing pressure inside the coat-hanger die. The temperature of the tempered rollers was 40 °C. The rotation speed was 0.4 m/min, and the draw speed was 1 m/min.

## 3. Results

### 3.1. Results of the Investigation of the PLAs and Thermally Expandable Microspheres

#### 3.1.1. The Molecular Weight Distribution of PLA

**Figure 1:**
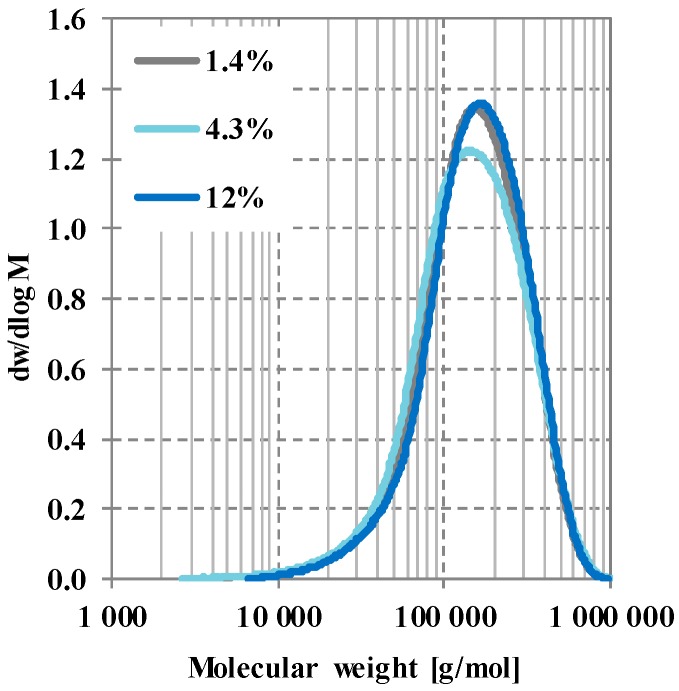
Molecular weight distribution of PLAs with different d-lactide content.

Gel permeation chromatography (GPC) analysis of polylactic acid (PLA) was used to confirm that there is no significant difference at the molecular level between the three types of PLA. The number average molecular weight of 4032D was 107296 g/mol, its weight average molecular weight was 183177 g/mol, and its polydispersity index (PDI) was 1.71. The $\bar{M_{n}}$ of 2003D was 100422 g/mol, its $\bar{M_{w}}$ was 180477 g/mol, while its PDI was 1.79. 4060D had an $\bar{M_{n}}$ of 116894 g/mol, an $\bar{M_{w}}$ of 191063 g/mol, and a PDI of 1.63 (Figure 1). Calculated by the Chi square test, χ^2^_4032D_2003D_(485) = 1.17, p = 0.95, χ^2^_4032D_4060D_(485) = 1.59, p = 0.95, χ^2^_2003D_4060D_(488) = 3.98, p = 0.95. The results indicate that with good approximation, the PLA raw materials differ significantly only in their d-lactide content.

#### 3.1.2. Rheological Tests of PLA

**Figure 2:**
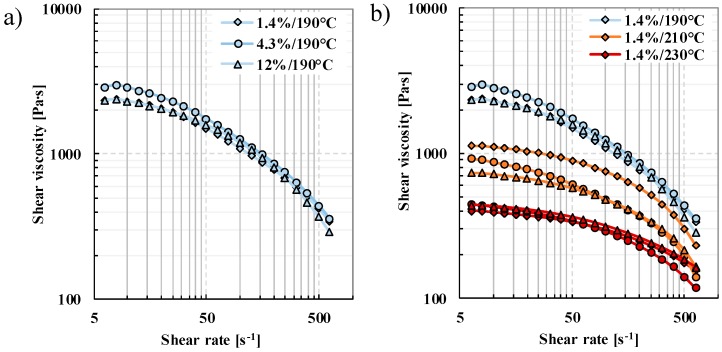
Shear viscosity of Ingeo 4032D (1.4%), 2003D (4.3%), and 4060D (12.0%) PLA (**a**) different d-lactide contents at 190 °C, (**b**) different temperatures (190 °C, 210 °C, and 230 °C).

The viscosity properties of raw PLA materials were investigated with an rotational rheometer. The effect of d-lactide content at 190 °C is shown in Figure 2a. It can be determined that at this temperature, shear viscosities of the PLA samples show a slight deviation relative to one another, they decrease with an increasing rate of shear (Figure 2b). The differences in the effect of d-lactide content tested at 210 °C are significant, as shown by the Chi square test. χ^2^_crit_ (1-p,r-1) = χ^2^_crit_(0.05, 20, upper tail) = 31.41, where all types of PLA compared to the others showed a p-value of 0.00000. At the lowest d-lactide content (1.4%), complex viscosity is the highest (1126 Pas), and viscosity decreases with the increase of d-lactide content. At a d-lactide content of 4.3%, complex viscosity is 921 Pas, while at 12.0%, it is 736 Pas. The reason for the decrease is that as the d-lactide content increases, the secondary forces in the polymeric material decrease [2]. This difference in viscosity almost vanishes at 230 °C. The temperature dependence of the viscosity of polylactic acid is shown in Figure 2b, which shows that increasing temperature decreases viscosity, which is expected [33].

#### 3.1.3. Isothermal Thermogravimetric Analysis of Expandable Microspheres

**Figure 3:**
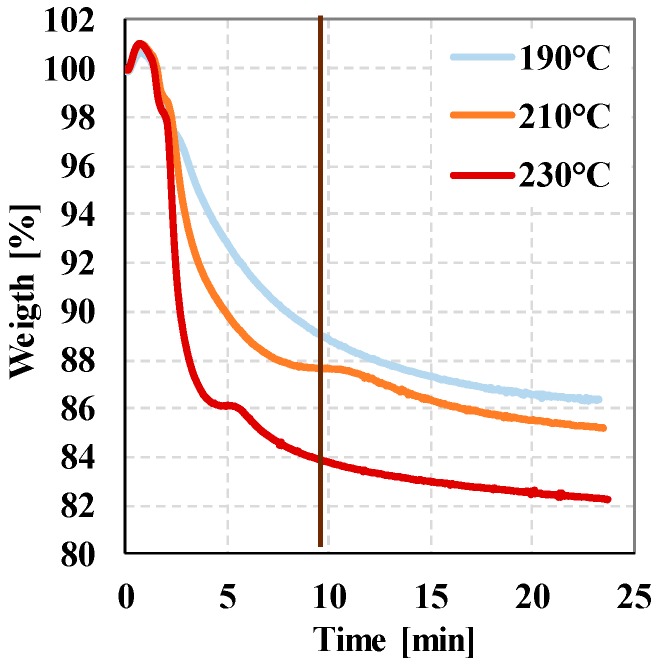
TGA (Thermogravimetric analysis) curves of expandable microspheres (from 10 min the measurement temperature is isothermal) weight loss as a function of temperature.

We performed TGA tests to determine the thermal properties of expandable microspheres. Figure 3 shows the weight loss of the foaming agent as a function of elapsed time. In the first minute of the test, a weight increase was observed due to the expansion of the foaming gas and the microspheres. At 190 °C, there was a 15% weight loss, while at 230 °C it was 18%. The results show that the weight loss of 13–18% is achieved more rapidly at higher temperatures—this weight loss is due to the expulsion of pentane from the microsphere (Table 2).

#### 3.1.4. Thermomechanical Analysis of EMS

**Figure 4:**
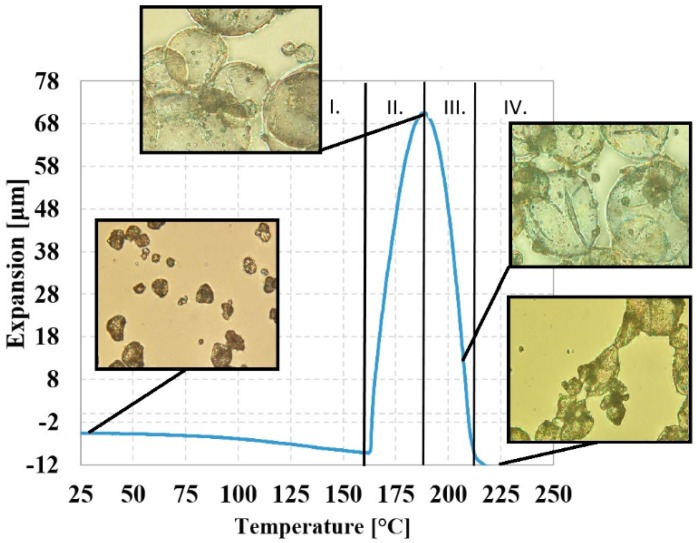
Analysis of the expansion of blowing agent Tracel G 6800 as a function of temperature, measured by Thermomechanical analysis (TMA), supported by optical microscopy pictures.

The expansion characteristic of the foaming agent was analyzed by thermo-mechanical analysis. Figure 4 shows the expansion of EMS due to temperature. Based on this, the EMS expansion-temperature curves can be divided into four different sections. Range I lasts from room temperature to the initial temperature of expansion (162.0 °C), where the average cell size of the EMS is 13.9 ± 7.7 µm at room temperature. Range II represents the growth section, which is the expansion phase with a maximum temperature of 187.9 °C and an expansion value of 70.48 μm. In Range III, expansion is reduced, shells were deformed, and the gas retention of the MMA/AN shell structure decreases. In Range IV, the complete failure of the EMS occurs.

### 3.2. Extrusion Foaming of Polylactic Acid with Expandable Microspheres

**Figure 5:**
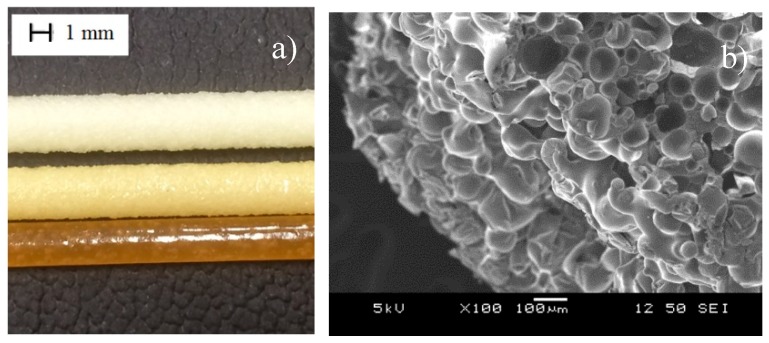
(**a**) The color of the produced foam structures at 190, 210, and 230 °C; (**b**) foam structure surface at 190 °C with 4.3% d-lactide content and with 8 wt% EMS.

During extrusion foaming it was found that the surface of the produced foams became increasingly yellow (Figure 5a) due to the thermal degradation of the MMA/AN material of the microspheres, which was confirmed by isothermal TGA tests. Additionally, we found that as we increased the amount of foaming agent, surface roughness increased proportionally, and the cells became visible on the surface of the specimen (Figure 5b), which is in agreement with previously reported results in the literature [34].

### 3.3. The Effect of EMS Amount

**Figure 6:**
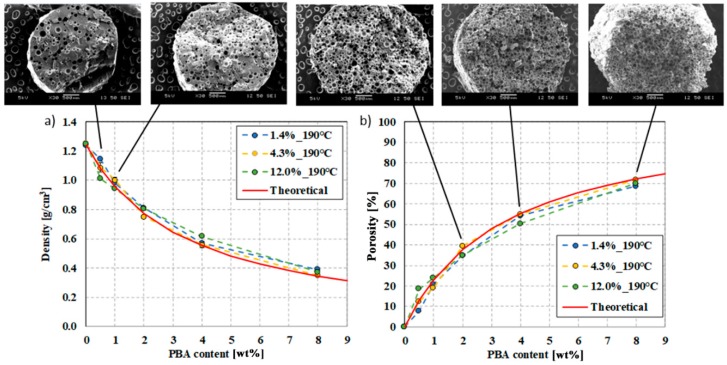
Density (**a**) and porosity (**b**) of samples produced at 190 °C as a function of foaming agent content (theoretically calculated density is shown with a red line).

One of the most characteristic properties of foam structures is their density. A red curve in Figure 6 shows theoretical density as a function of the amount of foaming agent, calculated with Equation (6). The exponential characteristic of the curve can be described with the following equation *ρ*(*x*)$\;=1.062\cdot e^{-\frac{x}{7.038}}+0.03$, R^2^= 0.947, where *x* is the percentage of foaming agent (Tracel G 6800). Figure 6 shows the density and porosity results of foamed samples (produced at 190 °C) as a function of the amount of EMS foaming agent between 0.5 wt% and 8 wt%. The density of PLA-based foam samples containing 1.4% (blue), 4.3% (yellow), 12.0% (green) d-lactide was almost the same in the investigated PBA content range. The lowest density of 0.35 g/cm^3^ was achieved with a processing temperature of 190 °C, a PBA content of 8 wt%. The highest achievable porosity is 72%. Depending on the amount of added foaming agent, density decrease approximates the theoretical density curve. When the PLA matrix contains 1.4% d-lactide, the descriptive equation is *ρ*(*x*)$\;=0.939\cdot e^{-\frac{x}{3.097}}+0.32$, R^2^ = 0.998. In the case of a d-lactide content of 4.3%, the descriptive equation is *ρ*$\left(x\right)=0.959\cdot e^{-\frac{x}{2.984}}+0.29$, R^2^ = 0.996, while in the case of 12.0% d-lactide, it is $\rho \left(x\right)0.886\cdot e^{-\frac{x}{3.359}}+0.31$, R^2^= 0.990.

**Figure 7:**
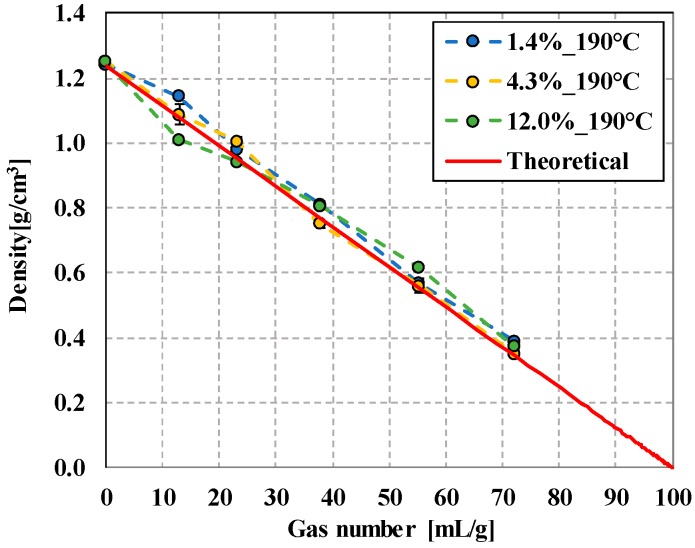
The density of samples prepared at 190 °C as a function of the gas number (theoretically calculated density is shown with a red line).

**Figure 8:**
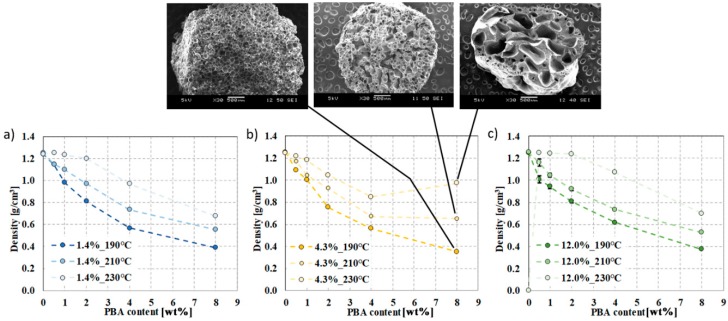
Density of foam structures at 190 °C, 210 °C, and 230 °C, with a d-lactide content of the matrix of (**a**) 1.4%, (**b**) 4.3%, (**c**) 12.0%.

The density as a function of the gas content of the foaming agent shows a linear trend. The descriptive equation is ρ(x)=1.24−0.0124x, R^2^ = 1, where *x* is the gas number. With the amount of gas added into the system, which is characterized by yielded gas, the density of the foamed rod samples decreases linearly, as in the theoretical case (Figure 7), which can be described with the equation ρ(x) = 1.24 − 0.0123*x*, R^2^=0.995. The lowest possible theoretical density is 0.346 g/cm^3^, which we successfully approached with a sample density of 0.35 g/cm^3^ (in the case of PLA with 4.3% d-lactide). The reason is that the expanding microspheres were intact in closed-cell foam structures during foam processing in PLAs of all three d-lactide contents; in all cases, the microspheres were able to expand sufficiently and form a homogeneous cell structure, which can be seen in the SEM images in Figure 8. The resulting maximum cell population density over the tested content (0.5–8.0wt%) is 10^11^ cells/cm^3^ at 8 wt%.

### 3.4. The Effect of Processing Temperature

Figure 8a–c shows the density of foam structures manufactured with a syntactic blowing agent as a function of the amount of foaming agent. As we previously pointed out, density as a function of the foaming agent exhibits an exponentially decreasing trend at 190 °C. The reason for density reduction is that at 190 °C, the expandable microspheres remained intact, forming a closed-cell foam structure, as the spheres were able to function as a cell nucleus. This trend is similar in the case of azodicarbonamide-foamed PVC systems, at low CBA content (<1 wt%) [35]. However, as production temperature is increased, density reduction as a function of the amount of foaming agent is lower at 210 °C and even lower at 230 °C. The results that we obtained correspond to the results of TMA and TGA, hence the maximum expansion of the expandable microspheres is at approximately 190 °C, and after that, the rate of expansion decreases. The reason for this effect is that the microspheres are unable to hold the pentane gas within the shell structure, and at higher temperatures, the microspheres collapsed.

Figure 8a shows a homogeneous cell structure produced at 190 °C. In contrast, samples containing 4 to 8 wt% foaming agent and produced at 210 °C show cells approximately the same diameter at 190 °C, but the foam structure also has larger amorphous cells. This indicates that the pentane has left the microspheres and was able to nucleate cells and cause cell growth due to the rupture of the shells. At 230 °C, the shells collapsed completely and the pentane gas is released from the spheres. Furthermore, the shape of these rod-shaped foams is not circular, but highly deformed.

### 3.5. The Effect of EMS Content and the d-Lactide Content of PLAs on Foam Morphology

**Figure 9:**
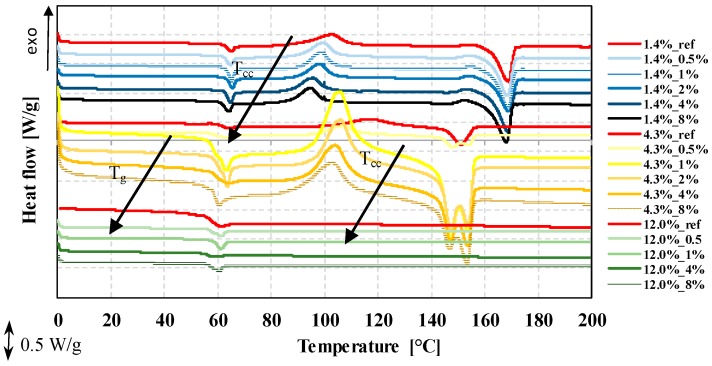
DSC (1st heating) curves of foams produced from three types of PLA with different d-lactide contents, processed at 190 °C.

The polymer morphology of the foamed samples was characterized by differential scanning calorimetry focusing on the first heating curve. Compared to the reference PLA (marked with a red line), the addition of the foaming agent affects cold crystallization temperature (T_cc_) (Figure 9). The PLA was able to crystallize at lower temperatures. Even at 0.5 wt% of EMS content, the cold crystallization temperature decreases markedly. The glass transition temperature (T_g_) also shows a slight decrease as a result of the addition of the foaming agent. Based on these, the presence of the foaming agent in the amorphous phase was able to increase segment movements. The presence of the foaming agent did not influence the enthalpy of the cold crystallization and crystalline melting processes or the crystalline fraction. The produced foam structures were nearly amorphous with a crystalline fraction of 0–5.5%, calculated with Equation (2).

The d-lactide content of PLA affects the crystallization tendency of PLA. Increasing d-lactide content decreases the crystallization tendency of the polymer. This trend of crystallization affects the stabilization phase of the foaming process. The crystalline fraction (X_cf_) which formed during manufacturing is related to the degree of expansion and porosity, i.e., it affects the degree of density reduction. The reason is that the polymer melt expands when it flows out of the die due to the decreasing pressure and semi-crystalline polymers can form a crystalline fraction on the outer parts, so the diffusivity of the polymer matrix is suddenly reduced. The crystalline regions cannot be tightly interconnected [2]. Foaming gases remain in a higher proportion in the polymer matrix and produce a higher rate of expansion.

**Figure 10:**
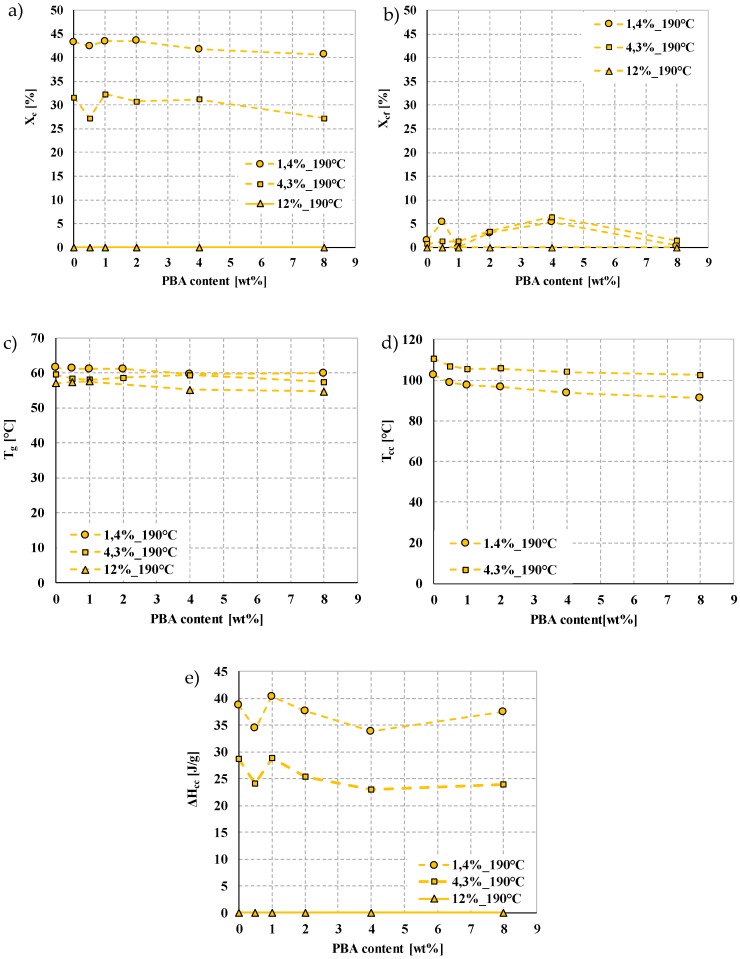
DSC (1st heating) results of foams produced from three types of PLAs with different d-lactide contents (**a**) crystalline fraction, (**b**) crystalline fraction during processing, (**c**) glass transition temperature, (**d**) cold crystallization temperature, (**e**) cold crystallization enthalpy at a processing temperature of 190 °C.

The DSC curves of the foam structures are shown in Figure 10. Based on the results, the crystalline proportion (Figure 10b) formed during production is highest with the lowest d-lactide (1.4%) PLA with the addition of 0.5 wt% or 4.0 wt% EMS. The results show that samples produced at 190 °C were typically amorphous with 0% crystalline fraction (at 12.0% d-lactide content) and a maximum crystallinity of 6.5% at 4.3% d-lactide content with 4 wt% EMS.

Furthermore, for all three d-lactide contents, the addition of the foaming agent to PLA decreased the peak crystallization temperature (T_cc_) (Figure 10d)—the PLA matrix is capable of crystallization at lower temperatures. Even 0.5 wt% EMS foaming agent reduces crystallization temperature considerably. The glass transition temperature also shows a slight decrease as a result of the addition of the foaming agent. The presence of the foaming agent (in any examined d-lactide content PLAs) did not affect the enthalpy of cold crystallization, the crystalline melting process, or the crystalline fraction formed.

### 3.6. Adapting Previous Results to Produce Foamed Sheet PLA Specimens

**Figure 11:**
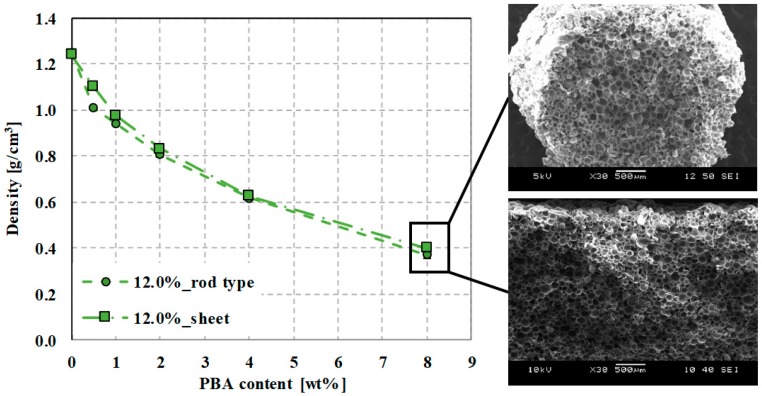
The density of produced rod and sheet samples at 190 °C as a function of foaming agent content.

Based on the results obtained from the production of rod-shaped specimens, we used temperature profile T1 to produce sheet foamed PLA specimens. The density of the produced foam sheets is shown in Figure 11. Both the manufactured sheet and rod samples show an exponential decrease in density. The lowest density we achieved with sheet specimens was 0.4 g/cm^3^ using 8 wt% PBA, and the highest porosity was 67.7%. Based on the SEM images, the average size of the cells formed is approximately the same at 8 wt% PBA content (rod sample: 93 μm and sheet sample: 91 μm). There is no evidence of cell deformation in the images; cells remained intact in both cases.

### 3.7. Mechanical Properties of Foams

#### 3.7.1. Compression Properties of Foam Structures

**Figure 12:**
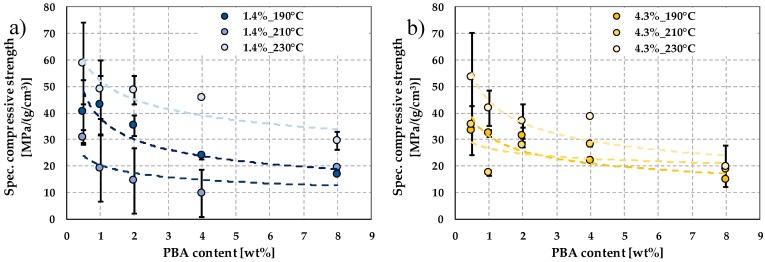

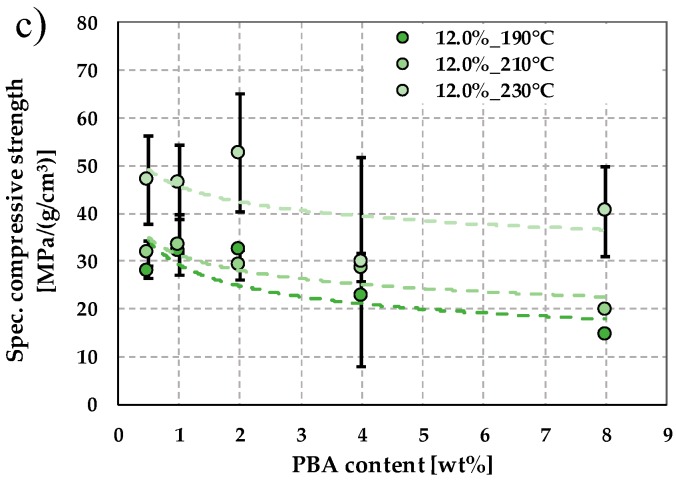
The specific compressive strength as a function of foaming agent content; specific compression strength of PLAs with (**a**) 1.4%, (**b**) 4.3%, and (**c**) 12.0% d-lactide content at different processing temperatures.

The quasi-static mechanical properties of the produced foams were tested by compression tests and characterized by density-specific compression strength. The specific compressive strength of the samples at a processing temperature of 190 °C (Figure 12a) shows a decreasing tendency as foaming agent content increases, and no significant difference can be observed between PLAs with different d-lactide contents.

As processing temperature increases to 210 °C and 230 °C, (Figure 12), compression strength tends to decrease as foaming agent content increases, but the standard deviation of specific compression strength increases. This tendency is due to the previously described inhomogeneous cell structure at 210 °C and 230 °C. The compressive strength of samples manufactured at 230 °C was difficult to measure because of the unfavorable, inhomogeneous foam structure with little expansion.

#### 3.7.2. Dynamic Mechanical Properties of Foam Structures

**Figure 13:**
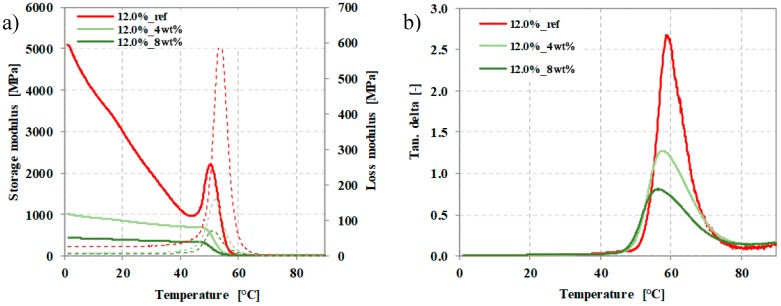
(**a**) Storage and loss moduli, (**b**) tanδ of the produced sheet foams as a function of temperature.

The produced foam sheet samples were suitable to carry out DMA measurements. We determined tanδ, storage and loss moduli based on dynamic mechanical analysis (Figure 13a,b). In addition, we evaluated the glass transition temperature at the maximum of the loss modulus peak. We also evaluated the storage modulus at room temperature (25 °C) and 10 °C above the glass transition temperature.

Table 3 contains the results from the DMA tests. The storage modulus of each sheet sample was greatly reduced above T_g_. Below T_g_ (at 25 °C), the foaming agents cause the storage modulus to decrease compared that of the non-foamed sample. In addition, the maximum value of the loss factor is reduced and lower than that of the non-foamed sheet sample. The glass transition temperature, calculated from the peak of the loss modulus shows a decreasing tendency as EMS content increases.

## 4. Conclusions

We produced extrusion foamed samples with a twin-screw extruder using a thermally expandable microsphere (EMS) type physical foaming agent with different amount (0.5, 1, 2, 4, 8 wt%) to investigate the effects of EMS content. We processed foams at three processing temperatures to demonstrate the effect of processing temperature on the EMS foaming agent. We also used three different types of PLA with different d-lactide contents to present the effect of d-lactide content on foaming.

We demonstrated that the reduction in density can be described with the following theoretical equation *ρ*(*x*)$\;=1.062\cdot e^{-\frac{x}{7.038}}+0.03$, R^2^ = 0.947, at 190 °C. Density as a function of the gas content of the foaming agent shows a linear trend, the descriptive equation of which is *ρ*(*x*) =1.24−0.0124x, R^2^ = 1. Therefore, the lowest possible theoretical density is 0.346 g/cm^3^, which we successfully approached with a sample density of 0.35 g/cm^3^ (in case of PLA with 4.3% d-lactide).

We also demonstrated that as production temperature increases, density reduction decreases at 210 °C and even more at 230 °C, compared to 190 °C. The reason for this effect is that the microspheres are unable to hold the pentane gas within the polymer shell structure, and at higher temperatures, they collapsed totally. This theory was supported by thermomechanical analysis. The results indicate that at higher temperatures, the pentane gas escapes from the microspheres and is able to nucleate cells and support cell growth due to the collapse of the polymer shell.

The morphological tests of the foamed samples show that the foaming agent has an effect on crystallization temperature (T_cc_) (Figure 9); the PLA crystallized at lower temperatures. Even 0.5 wt% EMS content lowered the temperature of cold crystallization significantly. The glass transition temperature (T_g_) also shows a slight decrease as a result of the foaming agent in the case of PLAs with all three different d-lactide contents.

We were also able to adapt our processing temperature profile for foamed sheet processing. The lowest density achieved with sheet specimens was 0.4 g/cm^3^ with the use of 8 wt% PBS and the highest porosity was 67.7%. We performed dynamic mechanical analysis of the foamed sheet samples. The presence of the foaming agent caused the storage modulus (at 25 °C) to decrease compared to the non-foamed sample. The storage modulus of each sheet sample was extremely reduced above T_g_ as well, and the maximum value of the loss factor was reduced, too. Glass transition temperature has a decreasing tendency with increasing EMS content.

## Figures and Tables

**Table 1:** Processing parameters of polylactic acid (PLA)-based foams via extrusion.

Zone Temperatures					Screw Rotation Speed	Notation
Z1	Z2	Z3	Z4	Z5	n	-
°C	°C	°C	°C	°C	1/min	-
155	160	175	190	190	10	T1
175	180	195	210	210	10	T2
195	200	215	230	230	10	T3

**Table 2:** The decomposition range of Tracel G 6800 EMS at different isothermal temperatures.

Sample_Temp	w_t=10 min_	w_t=23 min_
	%	%
Tracel G 6800_190 °C	88.8	86.3
Tracel G 6800_210 °C	87.6	85.2
Tracel G 6800_230 °C	83.8	82.3

**Table 3:** Dynamic properties of the produced sheet foams based on the DMA tests.

	Unit	12.0%_ref	12.0%_G6800_4wt%	12.0%_G6800_8wt%
T_(tanδMAX)_	°C	53.8	52.1	51.2
Storage modulus (at 25°C)	MPa	2476	812	384
Storage modulus (at T_g_+10°C)	MPa	8.5	7.9	10.6
